# Effects of Particulate Matter Education on Self-Care Knowledge Regarding Air Pollution, Symptom Changes, and Indoor Air Quality among Patients with Chronic Obstructive Pulmonary Disease

**DOI:** 10.3390/ijerph17114103

**Published:** 2020-06-09

**Authors:** Su-Er Guo, Miao-Ching Chi, Su-Lun Hwang, Chieh-Mo Lin, Yu-Ching Lin

**Affiliations:** 1 Department of Nursing and Graduate Institute of Nursing, College of Nursing, Chang Gung University of Science and Technology (CGUST), Chiayi County 613016, Taiwan; slhuang@mail.cgust.edu.tw; 2 Division of Pulmonary and Critical Care Medicine, Chang Gung Memorial Hospital, Chiayi County 613016, Taiwan; mcchi@mail.cgust.edu.tw (M.-C.C.); f124510714@adm.cgmh.org.tw (C.-M.L.); lin0927@adm.cgmh.org.tw (Y.-C.L.); 3 Chronic Diseases and Health Promotion Research Center, Chang Gung University of Science and Technology (CGUST), Chiayi County 613016, Taiwan; 4 Department of Safety Health and Environmental Engineering, Ming Chi University of Technology, New Taipei City 243303, Taiwan; 5 Department of Respiratory Care, Chang Gung University of Science and Technology, Chiayi County 613016, Taiwan; 6 Graduate Institute of Clinical Medical Sciences, College of Medicine, Chang Gung University, Taoyuan City 333324, Taiwan; 7 School of Medicine, Colledge of Medicine, Chang Gung University, Taoyuan City 333324, Taiwan

**Keywords:** particulate matter, chronic obstructive pulmonary disease, self-care management, health education

## Abstract

The burden of illness resulting from adverse environmental exposure is significant. Numerous studies have examined self-care behaviors among patients with chronic obstructive pulmonary disease (COPD), but seldom assess these behaviors in relation to air pollution. The study aims to examine the effects of particulate matter (PM) education on prevention and self-care knowledge regarding air pollution, symptom changes, and indoor PM concentration levels among patients with COPD. A longitudinal, quasi-experimental design using a generalized estimating equation examined the effectiveness of the education intervention. Participants were 63 patients with COPD, of whom only 25 received intervention. Levels of PM_2.5_ and PM_10_ decreased in the first-month follow-up in the experimental group. Improvement of knowledge and prevention regarding PM in the first and third months were also greater in the experimental group compared to the control. Regarding the COPD assessment test and physical domain scores, the experimental group exhibited a greater improvement in the first-month follow-up. Scores on the psychological domain significantly changed in the sixth-month follow-up. The PM education coordinated by nurses improved the health of participants, maintaining six-month effects. Further studies should evaluate the practice barriers and effects of health education on preventive self-care behaviors regarding indoor PM among patients with COPD.

## 1. Introduction

Fine particulate matter (PM_2.5_) in the atmosphere poses a risk to the lungs and alveolar region and is linked to cardiovascular diseases. Most previous studies have focused on the effects of particulate matter (PM) on cardiovascular disease or asthma [1,2,3,4]; however, the influence of PM on chronic obstructive pulmonary disease (COPD) is more severe than on the aforementioned diseases. COPD is the fourth leading cause of death in the United States and the seventh in Taiwan [5]. In addition, the health, social, and economic impact of COPD is an important issue worldwide. These effects tend to increase in a directly proportional way to the severity of the illness and to the number of acute exacerbations, especially those requiring hospitalization. In 2010, the global cost associated with COPD in the United States was $50 billion, of which 20 billion was the indirect costs and 30 billion was the direct costs [6]. In Taiwan, the average annual cost for each patient affected by COPD is approximately three million US dollars, but this does not include indirect costs [7]. 

Nevertheless, few studies have investigated the self-care behavior and medical resource utilization of COPD patients in the context of air pollution. Moreover, little is known about the contribution of health education on PM to self-care behaviors of COPD patients and disease symptoms. The association between COPD patients’ knowledge of PM, self-care behavior, PM levels, and disease prognosis have also not been explored. According to the mission and vision of International Council of Nurses (ICN), nurses are expected to serve communities, lead community health facilities, and deliver health care for all citizens. Therefore, we conducted a nurse-led longitudinal study, based on implementing a health education program including theoretical knowledge and evidence-based practice, with the aim of improving PM knowledge, self-management, health status, and indoor air quality (particularly PM_2.5_ and PM_10_ levels) for patients with COPD. 

### 1.1. Influence of PM on Respiratory Diseases

The size of particles has been directly linked to their potential for causing health problems. PM_10_ refers to particles with an aerodynamic diameter equivalent to or less than 10 μm, and fine PM (PM_2.5_) has a diameter of 2.5 μm or less. PM_2.5_ can enter the lungs more easily than PM_10_, so it has a greater impact on health. Wang used the Geographic Information System (GIS) to investigate differences in the spatial distribution of causes of death in central Taiwan, and estimated the concentrations and spatial distribution of PM and O_3_ in each area [8]. The results showed a strong correlation between mortality and PM, O_3_, and respiratory diseases for 20 causes of death [8]. 

Some studies consider that pollutants in the atmosphere, including NO_2_, O_3_, and PM, have an association with the occurrence of respiratory disease, and these pollutants damage and impair lung functioning [9,10]. In the United States, a study with 561 adult participants found that those who were exposed to high concentrations of air pollutants were more likely to have respiratory symptoms and lower lung function than those exposed to low concentrations of air pollutants [11]. Another systematic review found that concentrations of PM_10_ and PM_2.5_ were correlated with COPD, asthma, and other respiratory diseases, and the duration of the exposure to PM was also associated with the risk of mortality [12]. Furthermore, research in Taiwan demonstrated that ambient pollutants increase the occurrence of chronic respiratory diseases [13], and ambient PM and meteorological changes influence the risk of emergency treatment among elderly patients with COPD [14]. Hence, the harmful effect of ambient PM in terms of pulmonary and respiratory tract diseases cannot be ignored. 

### 1.2. Relation between PM, Symptom Changes, and Self-Care Behaviors in Patients with COPD

Several studies have demonstrated that PM_10_ and PM_2.5_ in the atmosphere are associated with mortality, acute exacerbation, and hospitalization rates of COPD patients [1,15,16,17,18]. Nevertheless, few studies have examined the symptom changes in COPD patients according to PM pollution, self-management of symptoms, and self-care behaviors. Self-care behavior has a buffering effect in the relationship between PM and acute exacerbation. If COPD patients are not aware of the link between symptom changes and PM concentration levels, they cannot engage in early prevention or pay attention to self-care regarding the avoidance of air pollution; thus, the risks of acute exacerbation, mortality, and hospitalization increase. Several studies have found that many cases of acute exacerbation among COPD patients at home are not reported or treated appropriately [19,20,21]. Although an unreported deterioration may be mild, research has established that not receiving appropriate treatment or care affects the health of patients with COPD [20,21]. 

Numerous studies have focused on self-care behaviors among patients with COPD, but most have concentrated on self-care behavior in general [22,23,24]. However, few studies have assessed self-care behaviors related to air pollution, patients’ low engagement, and difficulties relating to such behaviors. Poor self-care behavior leads to high morbidity and mortality and increases medical expenses [25]. Good self-care behavior can lower the severity of COPD and decrease the rate of hospitalization [26]. Research indicates that self-care behavior is crucial in managing COPD successfully. Effective self-care behavior helps to control the disease and avoid deterioration [27,28]. Considering that patients know and practice self-care behaviors to prevent deterioration as PM pollution increases or when symptoms occur, it is essential to improve patients’ knowledge about PM pollution and prevention of symptoms by providing a patient education program that increases self-care practices among patients with COPD. Therefore, investigating the effects of PM education on the symptoms and self-management of COPD patients is important.

## 2. Materials and Methods 

### 2.1. Study Design and Participants

A longitudinal, single blind, quasi-experimental design was used in the present study. All participants were recruited from outpatient clinics of the division of Pulmonary and Critical Care Medicine in a regional hospital in southwestern Taiwan and were divided into two groups by their willingness to participate: an experimental group and a control group. The experimental group received a single session of PM education, while the control group received usual care without PM education. The inclusion criteria were as follows: (1) aged 40 years or older; (2) COPD diagnosis by a chest physician; (3) moderate to very severe COPD according to the Global Initiative for Chronic Obstructive Lung Disease (GOLD) grades (30 < FEV_1_ predicted < 80%) [29]; and (4) ability to understand and communicate in Mandarin or Taiwanese. We excluded patients with any comorbidity or infection that may worsen because of PM, such as heart disease, asthma, tuberculosis, autoimmune diseases, or cancer.

### 2.2. Data Collection 

This study was approved by the Ethical Committee of the Human Body of the Chang Gung Medical Foundation (No. 1022417B). After explaining the purpose and procedure to potential participants, written informed consent was obtained from all those who were willing to take part in the study. After consent was obtained, home visits for data collection were arranged at four time points (baseline and one, three, and six months after baseline), in which interviewer-administered questionnaires were completed and environmental data collection took place through observation and technical measurements. 

The PM educational intervention was designed by the research team to provide knowledge and teach self-care regarding PM using education materials. It consisted of a single 45-min session that was completed by the experimental group after the pre-test (baseline). The single session education provided information on PM knowledge, PM level ranges, and prevention and self-management relating to abnormal PM levels. Providing information about the prevention of PM exposure entailed, for example, informing patients to pay attention to the news about PM, or identifying different colors that represented different concentration levels of PM. We assisted them by downloading the “Air Quality App” to access updated information on the air quality. We also taught them to identify the window closing/opening times based on the outdoor PM concentration according to this app. 

All home visits for both study groups included monitoring the home environment (PM_10_, PM_2.5_, temperature, humidity, and ventilation), evaluating lung function, and an interview using a structured questionnaire. The questionnaire included demographic characteristics, prevention and self-care knowledge regarding air pollution, dyspnea, and COPD symptoms. PM education intervention was delivered by one research assistant (RA) who is a master’s-level student in nursing science. All data collection and data input were performed by another RA in order to avoid bias by keeping the information blinded.

### 2.3. Measures

The study variables included the home environment (PM_10_, PM_2.5_, temperature, humidity, and ventilation), dyspnea assessed using the modified Medical Research Council (mMRC) dyspnea scale, COPD symptoms assessed using the COPD Assessment Test (CAT), prevention and self-care knowledge of PM, demographic characteristics (age, sex, education, COPD duration, living arrangement, smoking), and health status (Age-adjusted Charlson Comorbidity Index [ACCI] and lung function).

### 2.4. Environment Assessment

Environmental assessment included the measurement of PM_10_, PM_2.5_, temperature, humidity, and ventilation. Outdoor and indoor PM_2.5_ and PM_10_ concentration levels were detected using an aerosol spectrometer (Model TSI8532) in every home visit. We measured PM_2.5_ and PM_10_ in five areas: outside of the front door of the house, living room, bedroom, kitchen, and worship hall. Worship hall is a place that Taoists worship ancestors or gods with incense sticks in the morning every day and/or on the first and 15th day of each lunar month. The locations of the monitors in five places were consistent between the two groups: (1) Outside the front door of the house: 0.5 m outside from the front door of the house on the ground (street level); (2) living room: on the middle of the coffee table; (3) bedroom: on the bedside cabinet; (4) kitchen: on the middle of dinner table; and (5) worship hall: on the middle of the cabinet (at a height of about 100 cm). Each detection was performed three times for one minute each time and the best one of them was chosen. The calibration was performed before collecting the data to improve accuracy and reliability. Since PM concentrations are affected by meteorological influences, such as temperature, humidity, wind velocity, and indoor emissions, those variables were treated as confounders [30,31]. According to the 2012 Environment Protection Administration of Taiwan air quality standards [32], the daily mean or 24-h maximum of PM_10_ is 125 μg/m^3^, and that of PM_2.5_ is 35 μg/m^3^. 

### 2.5. COPD Symptoms

The modified Medical Research Council dyspnea scale (mMRC) and the Chinese version of the CAT were used to evaluate the symptoms of patients and assess changes at different time points. The mMRC is a well-known instrument with good reliability and validity to assess the syndrome of dyspnea in COPD patients; it uses a five-point scale from 0 to 4. Higher scores represent more breathlessness.

The CAT measures the impact of COPD on well-being and day-to-day activities that includes physical and psychological subscales, with a six point-Likert scale: 0 (I am very happy) to 5 (I am very sad). The score range is 0–40. The physical domain includes cough, sputum, chest tightness, activities of daily living (ADL), and breathlessness after walking up a hill or flight of stairs (five items). The psychological domain includes sleep quality, energy, and going outside with confidence (three items). A high CAT score denotes a poor perception of health status. The CAT has been well-known worldwide and a psychometric study using concurrent validity [33] and a systematic review study showed that it has good validity and reliability [34]. In recent years, the CAT has been widely used to evaluate the health status and severity of disease of COPD patients in Taiwan. Chai et al. [35] tested the validity and reliability of the CAT Chinese version using criterion-related validity and demonstrated that the scores of the Chinese version of CAT increased with the severity of lung function impairment, showing evidence that the scale has good validity. In addition, Cronbach’s alpha was 0.81, further demonstrating that it is a simple, reliable, and validated tool for patients with COPD. 

### 2.6. Self-Care Knowledge of PM

A questionnaire developed by the research team consisted of 12 items related to prevention and self-care knowledge regarding air pollution. The responses were coded as follows: “correct” responses were coded as “1”, and both “incorrect” and “I do not know” responses were coded as “0”. The possible scores range from 0 to 12; a higher score represents better awareness of prevention and self-care regarding air pollution. Regarding the validity, we invited three experts (a chest physician, an academic scholar in environmental science, and a head nurse in the public health center) to evaluate the content validity of this questionnaire. Its content validity using the scale-content validity index/average (SCVI/Ave) was 0.92, indicating good validity based on Shi’s study [36]; reliability was confirmed by a Cronbach’s alpha of 0.84. 

### 2.7. Age-Adjusted Charlson Comorbidity Index (ACCI)

The ACCI [37] is based on 19 diseases and is calculated as a weighted score from 0 (excluding any comorbid disease) to 6; and one point was added for each decade after 40 years of age (i.e., 41–50 coded = 1; 51–60 coded = 2; 61–70 coded = 3; ≥71 coded = 4.) A higher score indicates a higher illness severity [38].

### 2.8. Lung Function Test

Lung function was estimated by using a spirometer (MIR Spirolab II Pinyork, Taipei, Taiwan). The lung function tests are a group of tests that measure how well a patient’s lungs work. In this study, we used spirometry to measure a forced expiratory volume in one second (FEV_1_), and forced vital capacity (FVC) that reflects a patient’s lung function. Based on The Global Initiative for Chronic Obstructive Lung Disease (GOLD) [29]: Stage I (mild stage) are patients with FEV_1_/FVC < 70% and a predicted FEV_1_ of >80%; Stage II (moderate stage) with FEV_1_/FVC < 70%, and FEV_1_ 50–79%; Stage III (severe stage) with FEV_1_/FVC < 70%, FEV_1_ 30–49%; and Stage IV (very severe stage) with FEV_1_/FVC < 70%, and FEV_1_ < 30%.

### 2.9. Statistical Analysis

Baseline characteristics of the study participants were compared between the control and experimental groups using the chi-square test for categorical variables and independent-sample t-test for continuous variables. The intervention effect was assessed using a generalized estimating equation (GEE) which included main effects of intercept, covariates, study group (1 = intervention; 0 = control), and measurement (time points), and two-way interactions of “group × measurement”. The intervention effect was examined using a GEE model with a robust standard error and an exchangeable working correlation matrix adjusting for time [39]. The adjusted covariates varied for the different dependent variables and are detailed in the legend of Tables. More importantly, in PM concentrations we adjusted for meteorological influences including season, raining, temperature, humidity, and wind velocity based on previous studies [30,31]. The intervention effect was confirmed if the two-way interaction effect was significant. All tests were 2-tailed, and *p* < 0.05 was considered statistically significant. No adjustment for multiple testing (multiplicity) was made in this study. Data analyses were conducted using SPSS 25 (IBM SPSS Inc, Chicago, IL, USA).

## 3. Results

A total of 63 patients were enrolled, of whom 25 patients received the intervention and 38 did not. There were 21 and 30 patients in the intervention and control groups, respectively, who completed the measurements at all four time points. The remaining patients completed at least two measurements. Data were collected in similar seasons for the experimental and control groups and there was no statistically significant difference in the ratio between summer/winter (1.02 vs. 1.07, *p* = 0.84).

The mean age was 72.1 years (standard deviation [SD]: 8.2 years), with a range of 51–86 years. The mean COPD duration was 6.6 years (SD: 4.7 years), with a range of 1–20 years. The average FEV_1_ predicted value was 44% (SD: 19%). Table 1 lists the baseline characteristics of the patients in the experimental and control groups. Only a few patients were current smokers in both groups (20% vs. 27%, respectively); however, there was no statistically significant difference between them (*p* = 0.807). Patients in the experimental group had lower scores of knowledge regarding PM at baseline than the control patients (3.7 vs. 9.0; *p* < 0.001). Patients in the experimental group also had poorer lung functions, although the difference was not significant (FEV_1_ predicted, 40.1% vs. 49.4%; *p* = 0.076). No significant difference was observed between the two groups for the other baseline characteristics.

**Figure 1:**
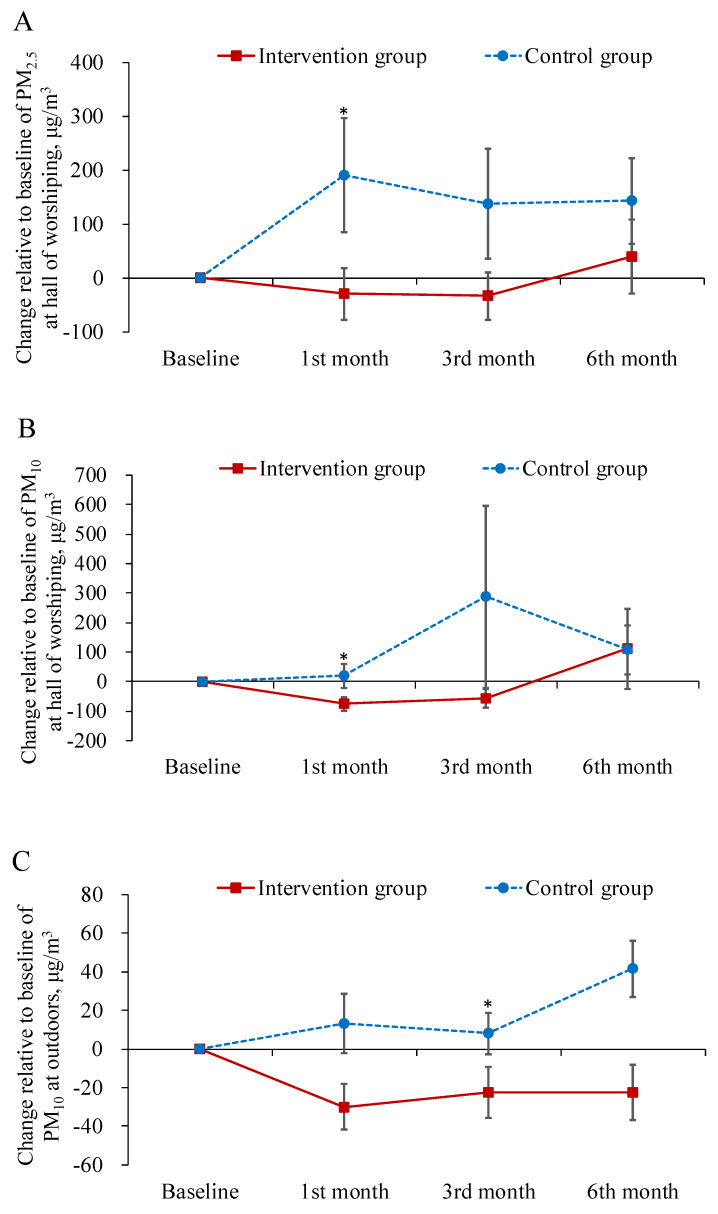
Mean difference from baseline to post-test and its standard error of particulate matter at each follow-ups in the intervention and control groups. * indicated the difference of the changed values from the baseline to a specific follow-up between the two groups was statistically significant.

Table 2 displays the descriptive statistics for the dependent variables in each study group across the time points of measurement. Table 3 summarizes the main results of the interaction effects in GEE analysis. The results demonstrated that the levels of both PM_2.5_ and PM_10_ in the worship hall decreased more from baseline to the first-month follow-up in the intervention group than in the control group (*B* = −243.49, −155.67 μg/m^3^; *p* < 0.05; Figure 1A,B). The levels of PM_10_ at outdoors decreased more from baseline to the three month follow-up in the intervention group as well (*B* = −18.32 μg/m^3^; *p* < 0.05; Figure 1C). 

**Figure 2:**
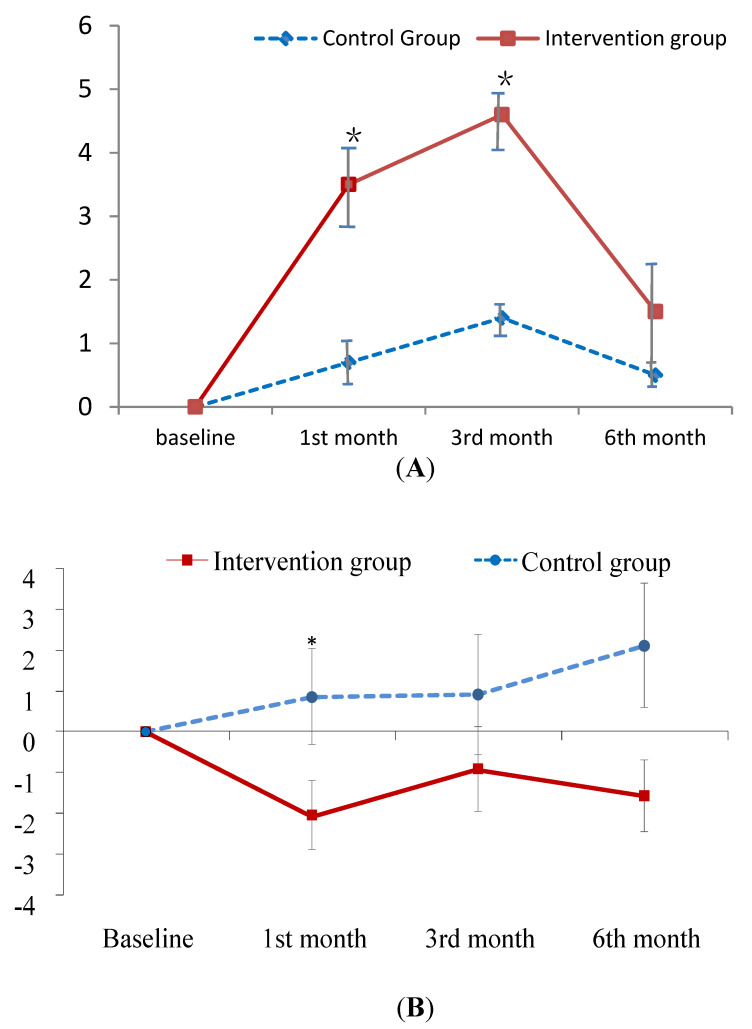

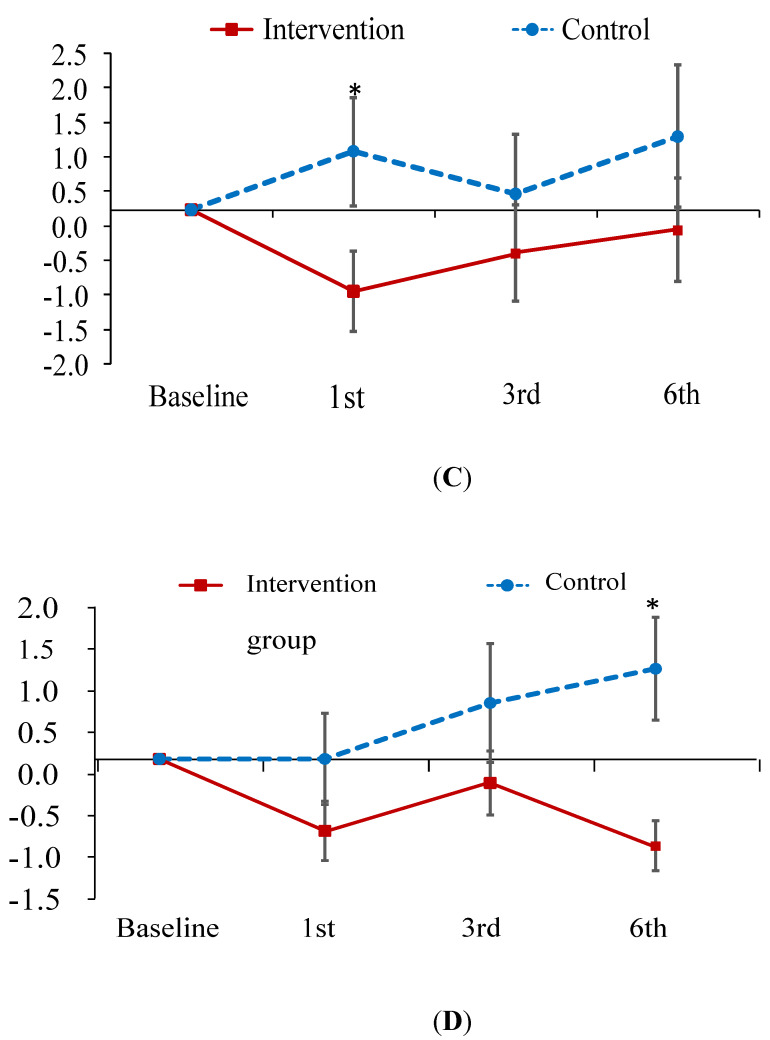
Mean difference from baseline to post-test and its standard error of knowledge on PM (**A**) and CAT score (**B**–**D**) at each follow-ups in the intervention and control groups. * indicates the difference of the changed values from the baseline to a specific follow-up between the two groups is statistically significant. (**A**) change relative baseline of knowledge on air pollution; (**B**) chage relative baseline of CAT score; (**C**) change relative to baseline of CAT physical score; (**D**) change relative to baseline of CAT psychological score.

The improvement in knowledge regarding PM at the first- and third-month follow-up was greater in the experimental group than in the control group (*B* = 2.73, 3.00; *p* < 0.001; Figure 2A). As for the total CAT and physical domain scores of CAT, patients in the experimental group exhibited a greater improvement at the first-month follow-up than those in the control group (*B* = −2.16, −1.54; *p* < 0.05; Figure 2B,C). In contrast, patients in the experimental group did not outperform the control patients on psychological domain scores until the sixth-month follow-up (*B* = −1.97; *p* < 0.05; Figure 2D). In addition, no treatment effect was observed in terms of FEV_1_ predicted value (Table 3). However, this is common finding since patient education has been found to have no effect on the lung function in several studies [40,41].

## 4. Discussion

To the best of our knowledge, this study is the first investigation to consider the influence of PM on COPD patients and the management of their symptoms. The results of the study revealed that the educational intervention was effective for COPD patients to improve their indoor environments regarding air pollution. In the first month after the intervention, both PM_2.5_ and PM_10_ levels in the patients’ worship hall decreased, which suggest that participants in the experimental group had more awareness and concerns about their environment than the control group did. Reducing the emission of burning incense in the worship hall was a particular challenge for the participants in this study. For most of the population of Taiwan, who are mainly Taoist or Buddhist, incense is associated with their religion and is used as a medium to communicate with their gods or ancestors; thus, it is an important trait of their religious behavior [42,43]. However, such emissions can produce PM and other chemical matters that can harm human health [44,45]. By increasing their knowledge of air pollution, participants changed their customs to improve their environments in the worship hall by reducing the number of incense sticks or by opening the windows while burning incense for worship. They became gradually more conscious of the risks of PM after the intervention and were willing to reduce their exposure to it, for example, by leaving the room after burning incense to prevent their disease from worsening. 

In addition, the decrease in exposure to environmental PM was associated with the change in CAT scores. In the first month, the total CAT scores as well as the physical scores of CAT improved, which could be due to patients’ improvements in taking avoidance measures to prevent their disease from worsening through exposure to PM. Adequate interventions and education effectively help COPD patients to manage their disease, enhancing their health condition and quality of life [46,47]. Moreover, the CAT score represents the respiratory condition of patients and reflects their quality of life; accordingly, the reduction in CAT scores was associated with the improvement of COPD symptoms after performing rehabilitation and receiving health education [48]. The improvement in CAT scores suggests that prevention knowledge regarding PM in this study led to the improvement of participants’ symptoms, such as chest tightness. However, the PM education intervention does not seem to significantly influence dyspnea symptoms using the mMRC scale. According to Table 2, the perceived dyspnea improved in the experimental group, but there was no significant difference in dyspnea symptom between the experimental and control groups. Is it possible that this result is because dyspnea is a subjective symptom and is based on the patients’ self-perception? Future research will need to control for treatment as a confounding variable and add more objective measurements, such as the six-minute walking test.

Whereas the physical status of participants improved in the first month after the intervention, the change in the psychological scores of CAT appeared at six months after the intervention. The psychological score of CAT represents patients’ perceptions of their mental health and confidence in the effect of outside activities on their health status/symptoms. COPD-related symptoms often cause anxiety or depression as well as social isolation because of the fear of exacerbation [49,50,51]; furthermore, depression is associated with the rate of readmission and exacerbation of symptoms among patients with COPD [51,52]. Another study found that activity-related dyspnea lowers the quality of life and increases depression, which also has an impact on the experience of and attitude toward COPD among patients [53]. On the other hand, when the PM in the environment decreased, the influence of physical symptoms on psychological aspects of COPD patients was not exhibited immediately in our study, but significantly appeared in the sixth month. This suggests that patients needed more time to perceive and realize the effects of PM education on their disease until experiencing alleviation of their physical symptoms and depressive emotions and participation in social activities. A study found that psychological well-being, including depression, anxiety, and quality of life, changed in COPD patients at a 10-week follow-up after an exercise rehabilitation intervention [49]. Psychological status improves as early as in the six-month follow-up, indicating PM education intervention is effective for COPD patients and corresponds to Hanania and O’Donnell’s proposal that education or health-related activities need to be implemented to improve COPD symptoms, mental health, and lower the risk of exacerbation [52].

The results show significant changes in prevention and self-care knowledge in the first- month and third- month follow-ups, but not in the sixth- month follow-up after the intervention. The reasons for no significant differences between the experimental and control groups in the sixth-month follow-up may be due to participants having a lower educational level (6.3 ± 3.8 years vs. 7.3 ± 3.6 years) and being older (72.8 ± 7.6 years vs. 71.6 ± 8.7 ) in the experimental group. Numerous studies [54,55] have documented that poor memory is associated with age; thus, providing supplemental material on PM is needed as a reminder for participants at every follow-up after the intervention, and this may have increased the knowledge of the intervention group in the sixth-month follow-up. Another reason may be that participants did not put the information from the education intervention into practice immediately after the intervention. According to the perspective of Csíkszentmihály [56], when individuals completely concentrate on implementing a mission or an activity without other distractions to achieve their goal, they will feel pleasure or excitement as they experience a state of flow which improves their life quality. To experience the state of flow, an interesting task with feedback and encouragement is necessary to arouse concentration and interest in a new challenge [56,57,58]. It is important to evoke the interest of participants, especially elderly patients, so that they memorize the information provided and practice the techniques learned in health education to improve their disease-related symptoms and life quality. Hence, providing information may be insufficient, and other factors need to be considered to help COPD patients to apply what they have learned.

Increasing patient understanding to implement this knowledge in daily life should be considered as a vital element affecting patients’ management of their disease and their indoor air quality. In particular, the elderly patients with COPD from rural areas in our study did not show a good response during the six-month follow-up because of their limited health education on PM and prevention; thus, repetitive education for a longer time would help them to increase their knowledge and self-care management [59]. A study in which four measurements were implemented every nine months for three years, indicated that knowledge of the disease did improve, but limited education did not show a significant change. Thus, a long-term investigation with repetitive exposure to education is needed to further examine the understanding and health-related behaviors of participants [59]. Furthermore, the education of patients should consider their different conditions or environments [60], so that health education interventions can correctly deliver information and enhance skills to improve the life quality of patients effectively. Moreover, Wang et al. observed that a 12-month follow-up assessing COPD patients’ status showed significant improvements in disease-related knowledge and self-management enhancing their quality of life [61]. Hence, more disease-related and PM-related techniques and practices are required for COPD patients to enhance their knowledge and implement better self-care behavior. A long-term follow-up (i.e., 6–12 months post intervention), may be needed to assess patients’ health status with treatment as confounders after an intervention, and continuously encourage them to maintain self-care practices. These practices include checking ambient air pollution via television, radio, and Internet app and following its recommended actions and health advisories, decreasing the frequency of incense burning and/or the duration of staying in the workship hall, drinking 2000–3000 cc water per day, and establishing regular sporting activities, to reduce the harmful effects of PM on their health. 

This study has limitations that should be acknowledged. First, we only measured PM concentrations within three-five minutes at each places, which may not represent actual exposure volume in long periods and might cause bias. However, we used relative changes of concentrations from time to time and collected data in a similar time period for both groups. Second, the PM concentration changes in the worship hall in the experimental group seems small although there were significant differences between the experimental and control group. Moreover, there were no significant differences in three- and six-month follow-ups between the two groups. More studies with randomized controlled trails and a large sample size need to be conducted in the future. Third, we did not analyze the composition of PM that influences cardiorespiratory health, which might vary. Several studies demonstrated that PM is toxic in terms of inflammatory, cytotoxic responses, lung cells, and the cardiovascular system which might cause lower respiratory symptoms, wheezing, and cardiovascular disease [62,63]. Future studies should analyze the composition of PM in order to identify the degree and trajectory of cardiorespiratory symptoms, which may provide references for nursing care and/or self-prevention within this population.

## 5. Conclusions

Overall, to the best of our knowledge, this study is the first investigation of its kind and bridges a gap in the existing literature by assessing the effect of PM education on the health environment and self-care management of disease among patients with COPD. The results revealed that the PM educational intervention was effective for patients with COPD to improve their environments in terms of air pollution, and enhanced their self-prevention knowledge and CAT scores. However, in order to increase their self-care behaviors for managing their condition and dyspnea when facing the threat of air pollution, further research needs to include long-term investigations with objective instruments and random allocation.

## Figures and Tables

**Table 1:** Baseline demographic and clinical characteristics of patients in the intervention and control groups (*n* = 63).

Variable	Intervention Group (*n* = 25)	Control Group (*n* = 38)	*p*
Male sex	22 (88.0)	35 (92.1)	0.674
Age, years	72.8 ± 7.6	71.6 ± 8.7	0.585
Education level, years	6.3 ± 3.8	7.3 ± 3.6	0.298
Living status			0.506
Living alone	5 (20.0)	5 (13.5)	
Living with someone	20 (80.0)	32 (86.5)	
Smoking			0.807
Never	3 (12.0)	5 (13.5)	
Quit	17 (68.0)	22 (59.5)	
Current	5 (20.0)	10 (27.0)	
Charlson Comorbidity Index	5.4 ± 1.5	5.1 ± 1.2	0.381
COPD duration, years	7.5 ± 4.6	6.0 ± 4.7	0.231
Burning incenses			1.000
None	3 (12.0)	4 (10.8)	
Occasionally	5 (20.0)	7 (18.9)	
Everyday	17 (68.0)	26 (70.3)	
Environmental status at baseline			
Raining	2 (8.0)	7 (18.9)	0.292
Outdoor temperature, °C	28.6 ± 5.9	27.3 ± 3.4	0.310
Outdoor humidity, %	68.0 ± 11.1	70.5 ± 11.1	0.393
Outdoor wind velocity, km/s.	0.47 ± 0.46	0.54 ± 0.47	0.551
Indoor temperature, °C	28.3 ± 5.3	27.4 ± 3.1	0.404
Indoor humidity, %	68.6 ± 9.3	68.9 ± 12.1	0.897
Indoor wind velocity, km/s.	0.19 ± 0.17	0.24 ± 0.19	0.301
Self-care knowledge of PM	3.7 ± 3.6	9.0 ± 1.7	<0.001
FEV_1_ predicted value, %	40.1 ± 15.1	49.4 ± 22.7	0.076

COPD, chronic obstructive pulmonary disease; PM, particulate matter FEV_1_, forced expiratory volume in one second. Data were presented as frequency (percentage) or mean ± standard deviation.

**Table 2:** Descriptive statistics of dependent variable in each study group across the measurements (*n* = 63).

Variable	Intervention Group (*n* = 25)				Control Group (*n* = 38)			
	Baseline	1st Month	3rd Month	6th Month	Baseline	1st Month	3rd Month	6th Month
PM_2.5_, μg/m^3^								
Outdoor	102.1 ± 71.4	76.8 ± 49.5	86.7 ± 77.3	88.7 ± 72.4	82.5 ± 60.3	85.9 ± 84.9	83.1 ± 61.7	111.5 ± 69.4
Living room	103.6 ± 75.5	115.0 ± 191.1	99.6 ± 98.2	91.7 ± 70.5	82.8 ± 58.3	82.3 ± 83.1	91.3 ± 73.2	100.7 ± 58.0
Kitchen	109.9 ± 78.8	116.2 ± 171.8	90.8 ± 77.3	94.3 ± 72.2	96.7 ± 69.2	89.2 ± 83.8	87.2 ± 64.5	116.9 ± 74.9
Bedroom	101.5 ± 78.1	113.1 ± 201.5	94.7 ± 75.0	87.4 ± 65.9	95.0 ± 114.5	91.8 ± 87.2	93.2 ± 67.5	106.7 ± 80.7
Worship hall	164.4 ± 199.6	135.2 ± 209.8	131.2 ± 200.9	204.5 ± 303.3	108.3 ± 82.2	299.0 ± 504.8	246.3 ± 503.7	252.1 ± 380.9
PM_10_, μg/m^3^								
Outdoor	112.3 ± 85.0	82.4 ± 55.6	89.9 ± 64.0	89.9 ± 70.3	88.2 ± 61.2	101.4 ± 86.1	96.4 ± 62.0	129.9 ± 83.6
Living room	106.7 ± 74.9	94.5 ± 84.1	97.4 ± 71.9	91.0 ± 69.6	88.7 ± 62.2	90.6 ± 76.6	91.0 ± 62.4	121.5 ± 72.9
Kitchen	117.2 ± 78.7	115.2 ± 152.8	97.0 ± 71.3	97.9 ± 73.7	104.7 ± 74.9	100.1 ± 84.0	93.6 ± 62.4	133.3 ± 86.7
Bedroom	107.5 ± 71.2	83.9 ± 73.9	96.5 ± 67.8	89.7 ± 69.9	86.3 ± 62.5	104.6 ± 97.0	96.7 ± 72.2	126.1 ± 82.5
Worship hall	187.2 ± 334.6	110.9 ± 98.3	129.5 ± 148.7	298.1 ± 607.3	168.4 ± 311.9	187.4 ± 197.2	455.5 ± 1516.9	275.7 ± 398.5
CAT scale								
Total	9.7 ± 6.0	7.7 ± 4.8	8.8 ± 5.9	8.1 ± 5.0	9.6 ± 6.2	10.4 ± 5.5	10.5 ± 7.2	11.7 ± 7.4
Physical	7.1 ± 3.7	5.9 ± 3.3	6.4 ± 4.0	6.8 ± 4.3	6.5 ± 4.2	7.4 ± 3.7	6.8 ± 4.2	7.6 ± 5.1
Psychological	2.6 ± 2.7	1.8 ± 2.0	2.4 ± 2.2	1.6 ± 1.7	3.0 ± 2.4	3.0 ± 2.6	3.7 ± 3.5	4.1 ± 3.0
mMRC	2.0 ± 1.5	1.7 ± 1.4	1.9 ± 1.4	1.6 ± 1.4	1.4 ± 1.0	1.4 ± 1.2	1.4 ± 1.1	1.3 ± 1.1
FEV_1_ predicted value, %	40.1 ± 15.1	41.0 ± 14.2	39.6 ± 16.6	39.2 ± 16.3	49.4 ± 22.7	45.8 ± 20.1	49.2 ± 21.0	51.3 ± 23.0
Self-care knowledge of PM	3.7 ± 3.6	7.2 ± 3.2	8.1 ± 2.1	5.2 ± 4.2	9.0 ± 1.7	9.7 ± 1.5	10.4 ± 1.1	9.5 ± 1.5

PM, particulate matter; CAT, COPD assessment test; mMRC, modified Medical Research Council dyspnea scale; FEV_1_, forced expiratory volume in one second. Data were presented as mean ± standard deviation.

**Table 3:** Summary of generalized estimating equation ^#^ (*n* = 63).

	Time	Interaction of Study Group by Time Points		
Outcome		1st Month	3rd Month	6th Month
PM_2.5_, μg/m^3^				
Outdoor ^a^		−33.42 (21.77)	−3.94 (12.77)	−23.87 (12.90)
Living room ^b^		20.11 (36.43)	4.94 (29.97)	−15.23 (26.93)
Kitchen ^b^		20.91 (34.78)	8.64 (28.18)	−23.52 (32.91)
Bedroom ^b^		26.71 (40.91)	18.32 (33.98)	4.67 (33.96)
Worship hall ^b^		−242.96 (106.82) *	−131.27 (118.01)	−58.73 (74.34)
PM_10_, μg/m^3^				
Outdoor ^c^		−29.15 (14.94)	−18.32 (8.89) *	−16.77 (8.60)
Living room ^b^		−8.15 (21.32)	4.91 (25.16)	−21.23 (14.67)
Kitchen ^b^		9.81 (31.71)	7.97 (29.13)	−22.56 (20.37)
Bedroom ^b^		−31.92 (22.65)	−1.64 (25.55)	−25.12 (17.12)
Worship Hall ^b^		−153.67 (75.99) *	−341.13 (267.71)	5.98 (100.24)
CAT scale ^d^				
Total		−2.17 (1.04) *	1.61 (1.42)	3.22 (1.66)
Physical		−1.55 (0.75) *	0.72 (0.86)	1.12 (1.03)
Psychological		0.63 (0.51)	0.89 (0.76)	−2.89 (0.82) *
mMRC ^d^		−0.49 (0.26)	−0.18 (0.26)	−0.30 (0.31)
FEV_1_ predicted value, % ^e^		3.73 (2.90)	−1.46 (3.34)	−4.67 (3.21)
Self-care knowledge of PM ^f^		2.73 (0.81) ***	3.00 (0.81) ***	1.06 (1.02)

PM, particulate matter; CAT, COPD assessment test; ^#^ Only interaction effects were reported for the sake of saving space; mMRC, modified Medical Research Council dyspnea scale; ^a^, adjusted for raining, outdoor temperature, outdoor humidity, outdoor wind velocity, indoor temperature, indoor humidity, indoor wind velocity, living room PM_2.5_, local PM monitoring data from EPA and self-care knowledge on PM at each time point; ^b^, adjusted for indoor temperature, indoor humidity, indoor wind velocity, local PM monitoring data from EPA, and self-care knowledge on PM at each time point; ^c^, adjusted for raining, outdoor temperature, outdoor humidity, outdoor wind velocity, indoor temperature, indoor humidity, indoor wind velocity, living room PM_10_, local PM monitoring data from EPA and self-care knowledge on air pollution at each time point; ^d^, adjusted for age, Charlson Comorbidity Index at baseline and FEV_1_ predicted value, six kinds of medication of COPD and self-care knowledge on PM at each time point; ^e^, adjusted for age sex, Charlson Comorbidity Index and six kinds of medication of COPD; ^f^, adjusted for age, Charlson Comorbidity Index, and self-care knowledge of PM at baseline; * *p* < 0.05, *** *p* < 0.001.
